# Repurposing risperidone as an anti-angiogenic agent for triple-negative breast cancer: a computational to *in ovo* investigation

**DOI:** 10.3389/fonc.2025.1645905

**Published:** 2025-10-28

**Authors:** Anisha Jain, Anil Kumar Belagal Motatis, Chandan Dharmashekar, Bhargav Shreevatsa, Siddesh VS, Monisha Srinivas, Sudhanva Muddenahalli Srinivasa, Ashwini P, M. N. Nagendra Prasad, Rafael Rosell, Jordi Codony-Servat, Shobith Rangappa, Muzaffar Iqbal, Kasim Sakran Abass, Raghavendra G. Amachawadi, Victor Stupin, Shiva Prasad Kollur, Chandan Shivamallu, Ekaterina Silina

**Affiliations:** ^1^ Department of Biotechnology, JSS Science and Technology University, Mysuru, Karnataka, India; ^2^ Adichunchanagiri Institute for Molecular Medicine, Adichunchanagiri Institute of Medical Sciences, Adichunchanagiri University, Nagara, Karnataka, India; ^3^ School of Natural Sciences, Adichunchanagiri University, Nagara, Karnataka, India; ^4^ Department of Microbiology, JSS Academy of Higher Education & Research, Mysuru, Karnataka, India; ^5^ Institute Germans Trias i Pujol, Badalona, Spain; ^6^ Instituto Oncológico Dr Rosell (IOR), Dexeus University Institute, Barcelona, Spain; ^7^ Pangaea Oncology, Dexeus University Hospital, Barcelona, Spain; ^8^ Department of Pharmaceutical Chemistry, College of Pharmacy, King Saud University, Riyadh, Saudi Arabia; ^9^ Department of Physiology, Biochemistry, and Pharmacology, College of Veterinary Medicine, University of Kirkuk, Kirkuk, Iraq; ^10^ Department of Clinical Sciences, College of Veterinary Medicine, Kansas State University, Manhattan, KS, United States; ^11^ Department of Hospital Surgery, Pirogov Russian National Research Medical University, Moscow, Russia; ^12^ School of Physical Sciences, Amrita Vishwa Vidyapeetham, Mysuru, Karnataka, India; ^13^ Department of Biotechnology and Bioinformatics, JSS Academy of Higher Education & Research, Mysuru, Karnataka, India; ^14^ Institute of Digital Biodesign and Modeling of Living Systems, I. M. Sechenov First Moscow State Medical University (Sechenov University), Moscow, Russia

**Keywords:** risperidone, triple-negative breast cancer, angiogenesis, drug repurposing, VEGFR2

## Abstract

**Introduction:**

Triple-negative breast cancer (TNBC) is a challenging subtype of breast cancer to treat because it lacks the expression of progesterone receptor (PR), estrogen receptor (ER), and human epidermal growth factor receptor 2 (HER2). A significant majority of deaths related to cancer are caused by tumor metastasis and angiogenesis. Vascular endothelial growth factor receptor 2 (VEGFR2) plays a significant role in angiogenesis. Instead of developing new molecules, drug repurposing, also known as repositioning, seeks innovative uses for outdated drugs or those that fail due to ineffectiveness.

**Methods:**

In this study, we performed high-throughput virtual screening of FDA approved drug library taken from Enamine bioactive collection targeting VEGFR proteins, and the top hit compounds analyzed by molecular dynamics simulations and MM-GBSA were considered for further *in vitro* analyses against human breast cancer cells, MDA-MB-231 and MDA-MB-468 cells followed by *in ovo* assay using the Chorioallantoic Membrane (CAM) model.

**Results:**

The results revealed that risperidone was effective against triple-negative breast cancer, with IC50 values ranging from 46.53 to 49.76 µM. The findings of our study demonstrated that risperidone, an antipsychotic drug, could successfully inhibit human breast cancer cells in silico, *in vitro* and *in ovo*.

**Discussion:**

We could prove that a structure-based drug repurposing approach is an effective strategy to produce a promising antiangiogenic repurposed drug that could also inhibit VEGFR2 in breast cancer. Although risperidone showed modest potency, its clinical availability and repurposing potential support further evaluation in preclinical and clinical settings.

## 1 Introduction

Breast cancer is a significant global health concern and a leading cause of cancer-related death among women worldwide. According to data from the Global Cancer Observatory (GLOBOCAN 2022), with an estimated 2.3 million new cases representing 11.6% of all cancer cases in 2022, breast cancer is the primary cause of cancer incidence worldwide among females. With approximately 6,66,103 deaths (6.9%), it ranks as the fourth most common cause of cancer-related mortality globally (1). With approximately 1,92,000 new cases (13.6%) and 98,300 deaths, breast cancer is the most common cancer in India (2).

Over the years, medical advancements have led to the development of better diagnostic and therapeutic approaches for breast cancer. However, despite these efforts, the prognosis for certain subtypes of breast cancer remains poor (3). Triple-negative breast cancers (TNBCs) are known to be negative for estrogen receptor, progesterone receptor or HER2 protein expression. The unusual metastatic patterns, aggressive nature, and lack of targeted therapy are the defining characteristics of TNBC. An estimated 170,000 instances are thought to be TNBC globally, accounting for approximately 10–20% of invasive breast cancer cases (4). TNBC is known for its aggressive nature, increased rate of recurrence, and tendency to spread to other parts of the body, making it one of the most challenging subtypes of breast cancer to treat (5, 6).

Angiogenesis is crucial to the growth and spread of breast cancer, according to several reports. Angiogenesis plays a key role in the development of invasive cancer from hyperplastic *in situ* epithelium (7). Based on the evaluations from both experimental and clinical investigations, vascular endothelial growth factor (VEGF) is the most significant angiogenic factor that has been shown to be significant in breast cancer. Potential strategies for treating TNBC include the use of EGFR tyrosine kinases, poly(ADP–ribose) polymerase 1, and VEGF targeting (8, 9). In VEGF signaling, vascular endothelial growth factor receptor-2 (VEGFR2) is a master node and a receptor tyrosine kinase. VEGFR-2 is composed of two tyrosine kinase domains inside the intracellular region, a transmembrane domain, and seven immunoglobulin-like domains within the extracellular region (10, 11). Each VEGFR family has distinct traits. Among them, VEGFR2 has been found to be a viable target for tumor therapy (12).

Studies have also shown that targeting VEGFR 1 and 2 could be a potential therapeutic approach for TNBC. In preclinical models, the inhibition of VEGFR 1 and 2 has been shown to decrease tumor growth and metastasis. Several clinical trials are currently underway to evaluate the safety and efficacy of VEGFR inhibitors in TNBC (13, 14). Antiangiogenic drugs have been studied in metastatic breast cancer clinical trials, with rather contentious results, particularly in regard to TNBC (15, 16). The use of VEGFR2 inhibitors could reverse the propensity of breast cancer cells for endothelial migration and angiogenesis (17, 18). Based on a previous report, TNBC cells produce VEGFR2 and secrete VEGF, which both autocrinely stimulates endothelial cells that express high amounts of VEGFR2 and, in parallel, promotes the proliferation of TNBC cells that express VEGFR2 (19–21). Despite advances in systemic therapy, the absence of an effective, well-tolerated anti-angiogenic strategy for triple-negative breast cancer remains a major unmet need. Identifying safe, clinically accessible agents that can disrupt tumor angiogenesis is therefore a key research priority. Targeting VEGFR2 can be a promising therapeutic approach for TNBC, and the current research is aimed at developing more effective therapies to improve outcomes for patients with this aggressive subtype of breast cancer (14, 22). Recent reviews emphasize that integrating anti-angiogenic approaches with other therapeutic modalities may help overcome these limitations and improve outcomes. Building on this context, our study investigates an alternative strategy to inhibit angiogenesis and restrain TNBC growth.

Drug repositioning or repurposing is the cause of approximately 30% of the drugs and vaccines that the U.S. Food and Drug Administration recently approved. Compared with developing a new drug from scratch, drug repositioning is faster, more cost-effective, and carries less risk to both companies and patients (23–26). Structure-based drug repurposing is a computational approach that uses the three-dimensional structure of a protein to identify new uses for existing drugs or drug candidates. This approach involves analyzing the protein structure and comparing it with a database of known drugs to identify compounds that may bind to the protein and modulate its activity. The process of structure-based drug repurposing typically involves several steps, including protein structure determination, virtual screening, hit identification, hit-to-lead optimization, and preclinical testing (25–27). This study aimed to evaluate the repurposing potential of an FDA approved drug compound for its potential to inhibit angiogenesis and suppress TNBC cell growth using an integrated approach combining computational modelling, *in-vitro* assays, and an *in-ovo* CAM model.

## 2 Materials and methods

### 2.1 Structure-based virtual screening

Virtual screening involves the use of computational tools to dock potential drug candidates onto the protein structure and identify compounds that have a high likelihood of binding to the target (25). The *in silico* analysis for the study was performed *via* the Schrödinger Suite 2023 (28). It is a software for chemical and biological applications that provides a number of tools that make it easier to investigate chemical system structures, reactivities, and characteristics, as well as to produce spectacular, high-performance molecular visuals for presenting structural findings (29).

### 2.2 Protein selection and preparation

The three-dimensional crystal structures of VEGFR I (PDB ID: 3HNG) (30) and the VEGFR 2 kinase domain (PDB ID: 4AG8) (31) were retrieved from the RCSB protein data bank (PDB) (**Supplementary Figure S1** **),** with resolution values of 2.70 Å and 1.95 Å, respectively. These structures were prepared via the protein preparation wizard Maestro v13.3 to fix all the problems in the existing structures. The structures were pre-processed by assigning bond orders, adding missing hydrogens and disulfide bonds, filling in missing loops using Prime module, removing water molecules, ions/metals and other co-crystalized ligands followed by hydrogen bond optimization to improvise charge-charge interaction and hydrogen bonding. During this process, using the predicted pKa values, the pH was adjusted to 7 ± 0.5. These pre-processed structures were further used for the in silico analysis (27).

### 2.3 Ligand library preparation

A library of FDA-approved drugs in SDF format was retrieved from the Enamine Bioactive collection ((https://enamine.net/compound-libraries/bioactive-libraries/fda-approved-drugs-collection)-accessed on 10-01-2024) and imported into the workspace of Maestro. To prepare the library for high-throughput virtual screening (HTVS), the LigPrep tool was used. The criteria for ligand preparation included the OPLS_3_ force field, the generation of one conformer for each ligand and a pH of 7 (± 2) to generate possible ionization states (27).

### 2.4 Binding site prediction and grid receptor generation

The binding sites of both proteins were retrieved *via* the SiteMap tool of the Schrödinger Suite. The top-ranked potential binding site (site 1) with at least 15 site points per reported site was selected in the receptor grid generation tool of the suite to generate a grid surrounding the binding site amino acid residues for both proteins, which generated grid zip files as the output. All the grids were generated with a scaling factor of 1 and partial charge cut-off of 0.25. The dimensions of the grid box for VEGFR1 were 80 Å (x, y and z axis) and that of VEGFR2 were 72 Å (25).

### 2.5 High-throughput virtual screening

The ligand output file was then subjected to a structure-based virtual screening procedure utilizing the Schrodinger suite’s Glide module. In the ligand docking panel, the receptor grid file was imported, and the HTVS precision followed by SP (standard precision) mode for top hits was selected with a single pose per ligand as the output. Docking was performed with the OPLS3e force field. The scaling factor of van der Waals radii was set at 0.80 with a partial charge cutoff at 0.15 (25, 28).

### 2.6 Molecular dynamics simulation

The compounds were then filtered on the basis of the Glide score (Kcal/mol), protein–ligand nonbonded interactions, and ligand–active site complementarity and on the basis of a review of the literature. Based on the average glide score, of the two VEGFR proteins, one was selected for further analysis. The top two complexes were further subjected to MD simulations *via* the Desmond module of the Schrödinger Suite 2022–3 to analyze the intermolecular interactions and the stability of the complex at various time scales. To build the system, an orthorhombic shaped box of 14 Å distance embedded with TIP3P solvent model was generated, and counterions were added to neutralize the system. The obtained model system was then loaded into the molecular dynamics work panel, and the simulation run time was set at 100 ns. Throughout the simulation time, the docked complexes were assessed at pH 7.0 ± 0.2, and NPT ensemble class (Nose-Hoover thermostat and Martyna-Tobias-Klein barostat) to preserve the same pressure and temperature throughout the run at 1.01325 bar and 310 K, respectively After the completion of the simulation run, the complexes were analyzed *via* the simulation interaction diagram panel of the Desmond module (32, 33).

### 2.7 Molecular mechanics generalized born surface area analysis

To determine the strength of the interactions and validate the docking results, MM-GBSA was used to estimate the ligand-binding free energy (ΔG_bind_). The protein–ligand docked complexes were subjected to the Schrodinger suite’s Prime module with the default setting for these calculations. These calculations were performed on the top two complexes. The best compound was then taken for *in vitro* analysis (32, 33).

### 2.8 Cell lines and culture conditions

All the chemicals and reagents used in the present study were procured from Sigma Chemical Co. (St. Louis, MO) and MP Biomedicals (Fountain parkway solon, Ohio, USA). The human triple-negative breast cancer cell lines MDA-MB-231 and MDA-MB-468 were purchased from the National Centre for Cell Sciences (NCCS), Pune, India. The human normal kidney cell line HEK-293 was generously gifted by Prof. Sathees C. Raghavan, Dept of Biochemistry, Indian Institute of Sciences (IISc), Bengaluru. The cells were cultured in RPMI 1640 and high-glucose DMEM with 2 mM L-glutamine (Thermo Fisher Scientific, Inc.; Waltham, MA, USA) supplemented with 10% FBS (Gibco; Grand Island, NY, USA). The cells were cultured in a humidified incubator with 5% CO_2_ at 37 °C. Cells were used between passages 5–15 to ensure consistent growth characteristics. The drug was purchased from a pharmaceutical store.

### 2.9 Preparative high-performance liquid chromatography separation

The separation and isolation of the risperidone compound from the Risperdal tablet were performed *via* a water HPLC-XTerra MS C18 OBD Prep column (125 Å, 10 µm, 10 mm X 250 mm). The mobile phase used for separation was 0.2% formic acid buffer and acetonitrile. The injection volume was 20 µl, the flow rate was 10.56 ml/min, the ratio of the passive splitter was 1/3500, the flow rate of the compensation solvent was 1.5 ml/min, and the solvent was acetonitrile, water, or formic acid (50:50:0.2 v/v/v). For the present study, the risperidone compound was isolated *via* MS, which was set on the basis of the selected ion monitoring (SIM) mode. The MS parameters were as follows: the temperature was 100 °C, the capillary voltage was 3 kV, the cone voltage was set to 35 V, the desolvation gas and cone flow rates were set to 450 L/h and 20 L/h (34–36). Although pharmaceutical-grade risperidone standards are available, we isolated risperidone from commercial tablets to eliminate excipients that could interfere with biological assays.

### 2.10 MTT and Trypan blue dye exclusion assays

The MTT and Trypan blue dye exclusion assays were performed as described previously (37). Seeded cells were treated with increasing concentrations of risperidone (10, 25, 50, and 100 μM), and DMSO was used as the negative control(≤ 0.5% (v/v)). After incubation, the cells were subjected to MTT and Trypan blue dye assays. The insoluble formazan crystals were solubilized with 100 μL of DMSO and subjected to spectrophotometric absorbance at 570 nm using a Tecan Microplate Reader (Tecan Instruments, Switzerland). The Trypan blue-treated cells were counted *via* Thermo Fisher Countess IIIFL to calculate the percentages of live and dead cells. The half-maximal inhibitory concentrations (IC50s) were determined *via* GraphPad Prism 9.0 software (GraphPad Software Inc., San Diego, CA, USA) with the 4-parameter logistic function standard curve analysis for dose–response. Error bars were calculated on the basis of a minimum of three independent experiments, and the data are represented as a histogram.

### 2.11 Hoechst/PI staining

The Hoechst/PI double-staining assay was performed as described previously (38). The seeded MDA-MB-468 cells were allowed to attach overnight and then treated with increasing concentrations of risperidone. After incubation for 48 h, the cells were subjected to Hoechst/PI double staining.

### 2.12 Colony formation assay

CFA was performed as described previously (39). Briefly, MDA-MB-468 cells were seeded into a 6-well plate and allowed to attach overnight. They were then treated with increasing concentrations of risperidone and incubated for two weeks with intermittent media changes. After the incubation period, the cells were washed with PBS, fixed in methanol:acetic acid, stained with crystal violet, allowed to dry, and imaged. Colonies >50 cells were counted manually and validated with ImageJ.

### 2.13 Mitochondrial membrane potential assay

The MMP assay was performed as described previously (39). MDA-MB-468 cells were seeded at 1 × 10^6^ cells per 96-well plate, allowed to grow overnight, and then treated with different concentrations of the drug. After 24 h of incubation, the cells were subjected to the MMP assay according to the manufacturer’s protocol (MAK159, Sigma Aldrich). Spectrophotometric absorbance of the plate was recorded at (λex = 490/λem = 525 nm) and (λex = 540/λem = 590 nm) for ratio analysis using Tecan Microplate Reader (Tecan Instruments, Switzerland). The cells were subsequently centrifuged at 800 rpm and imaged *via* an Olympus CKX53 inverted fluorescence microscope (19).

### 2.14 Chorioallantoic membrane assay

The CAM assay is considered a refinement model, and according to CPCSEA (India) and EU Directive 2010/63/EU as well as National Institutes of Health, USA, mandated that a chick embryo that has not reached the 14th day of its gestation period would not experience pain and can therefore be used for experimentation without any ethical restrictions or prior protocol approval (40). The antiangiogenic effect of risperidone was evaluated *via* the CAM model. Fertilized eggs were obtained from a hatchery in Mysuru, Karnataka, India. The eggs were incubated for 72 h at 37–39 °C and 50%–60% humidity, which was maintained by adding sterile distilled water to the incubator. After 72 h of incubation, the eggs were sterilized with 70% ethanol. Day 4 involved making a square hole in the outer shell and using a 1 ml pipette to extract 1–1.5 ml of albumin, which allowed the CAM membrane to separate. Next, a sterile blank filter disk with 50 μg/ml risperidone, 10 ng/mL VEGF, or the vehicle control was placed on top of the CAM under sterile conditions. After that, the window was covered with parafilm, and the eggs were incubated for 48 h at 37 °C and 60% humidity. The CAM images were captured with a camera. The images were loaded into AngioTool software, and the average total length and number of branching sites/junctions in the blood vessels were measured. Analysis was conducted *via* the following parameters: vessel diameter and intensity thresholds were set at 30 and 255, removal of small particles at 200 (41, 42).

### 2.15 Statistical analysis

Each experiment was conducted in triplicates. The data was presented in the form of mean ± standard deviation. Comparisons between two groups were evaluated with an unpaired, two-tailed Student’s t-test. For analyses involving more than two groups, a one-way analysis of variance (ANOVA) followed by Tukey’s *post-hoc* test was applied. A value of p < 0.05 was considered statistically significant. Data was analyzed by GraphPad prism software (GraphPad Software Inc., San Diego, CA, USA).

## 3 Results

### 3.1 High-throughput virtual screening analysis

A graphical representation of the glide scores is given in **Supplementary Figure S2**. In comparison, more compounds had scores between -8 and -10 when docked with VEGFR2 than when docked with VEGFR1. The more negative the docking score is, the better and greater the binding affinity is because Glide docking techniques calculate a docking score that is related to the free energy of binding of a ligand to a receptor. Hence, further analysis of VEGFR2 was conducted.

**Figure 1:**
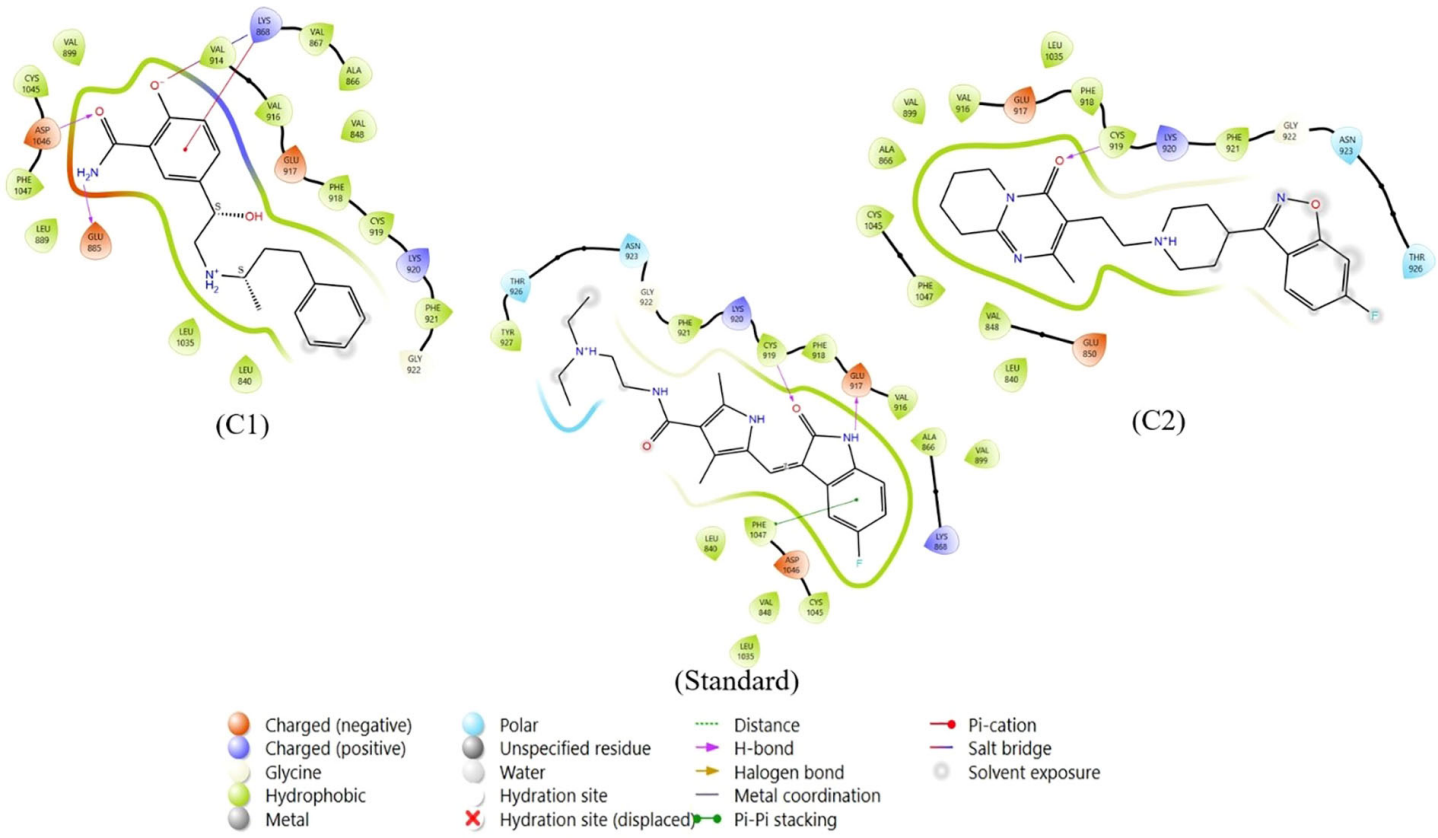
Two-dimensional protein–ligand interactions of VEGFR2 docked to repurposed compounds: the compound labetalol (C1), the compound risperidone (C2) and the standard sunitinib.

After thorough filtering of compounds on the basis of Lipinski’s rule of five, the glide score, nonbonded interactions and a review of the literature on existing inhibitors of VEGFR, two compounds, labetalol and risperidone, were considered for further analysis and compared withSunitinib, a standard inhibitor of VEGFR. **Table 1** represents the Glide scores (Kcal/mol) of selected docked complexes of VEGFR1 and VEGFR2, and **Figure 1** represents the 2D protein–ligand interactions of the VEGFR2 docked complexes in comparison with the standard. The compound labetalol interacts with amino acid residues LYS868 (Pi cation), GLU885 (H-bond), and ASP1046 (H-bond), whereas the compound risperidone binds to residue CYS919 (H-bond) in comparison with the standards, which bind to GLU917 (H-bond), CYS919 (H-bond), and PHE1047 (Pi–Pi stacking).

**Table 1:** Glide scores (kcal/mol) of the docked complexes.

Small molecules	Glide scores (Kcal/mol) of VEGFR1	Glide scores (Kcal/mol) of VEGFR2
Labetalol (C1)	-8.07	-8.45
Risperidone (C2)	-8.44	-9.56
Standard	-8.21	-9.32

### 3.2 MD simulation interaction analysis

**Figure 2:**
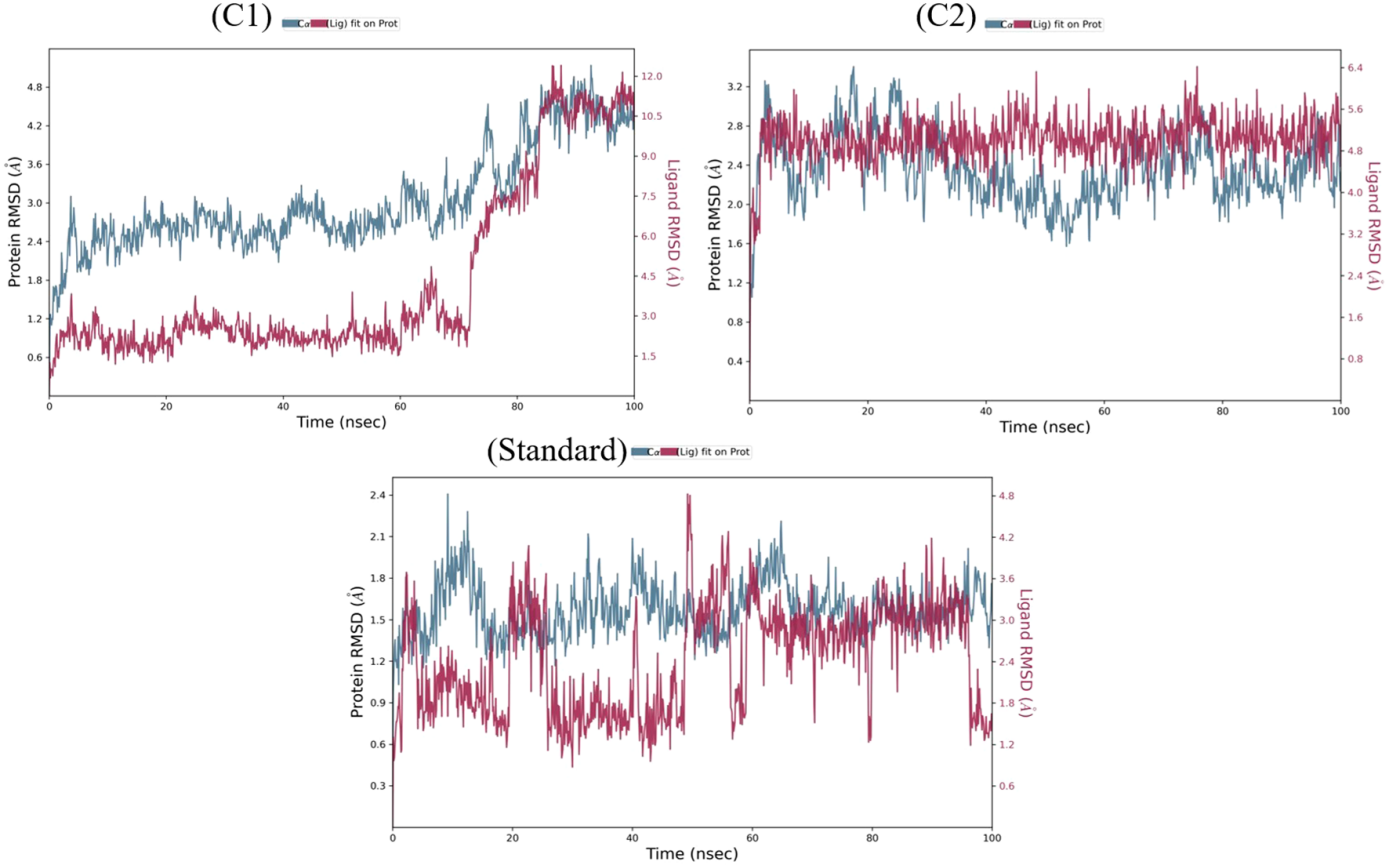
The RMSD plots of labetalol (C1), risperidone (C2) and the standard in complex with VEGFR2.

The two repurposed compounds and the standard inhibitor were subjected to simulations lasting 100 ns and the resulting trajectories were examined *via* a simulation interaction diagram panel to determine de*via*tion (**Figure 2**), fluctuation (**Supplementary Figure S3**) and intermolecular interaction (**Supplementary Figure S4**) analyses. The deviation in the protein backbone throughout the course of the simulation period of 100 ns is calculated *via* the root mean square deviation (or RMSD) value. The RMSD graphs (**Figure 2**) clearly revealed that the VEGFR2-risperidone complex was more stable throughout the experimental period than was VEGFR2-labetalol. The RMSDs of the compound risperidone fluctuated, with a maximum value of 6.4 Å at 48 ns and 75 ns and slight fluctuations ranging from 4.8 to 5.7 Å. The RMSDs of the standard compound deviated more from 0 to 20 ns, and more fluctuations were observed throughout the 100 ns time frame. The local alterations along the protein chain can be characterized *via* the root mean square fluctuation (RMSF) (**Supplementary Figure S3**). Beta-strand and alpha-helical sections are distinguished by their respective red and blue colors, and the vertical green bars indicate amino acid binding residues that interact with the ligand. In all three complexes, large peaks were observed that were not in contact with the ligands. The protein-ligand interaction fraction determines how long the specific interaction is maintained throughout the simulation time (**Supplementary Figure S4**). For the labetalol complex, LYS 868 maintained up to 20% hydrogen bonding, followed by hydrophobic interactions (5%), ionic interactions (50%), and water bridges (50%). The ASP 1046 residue exhibited maximum hydrogen bonding of up to 100%. The residue CYS 919 exhibited the most hydrogen bonding throughout the simulation period for the risperidone complex, whereas in the standard, the residues were GLU 917 and CYS 919.

### 3.3 MM-GBSA analysis

**Figure 3:**
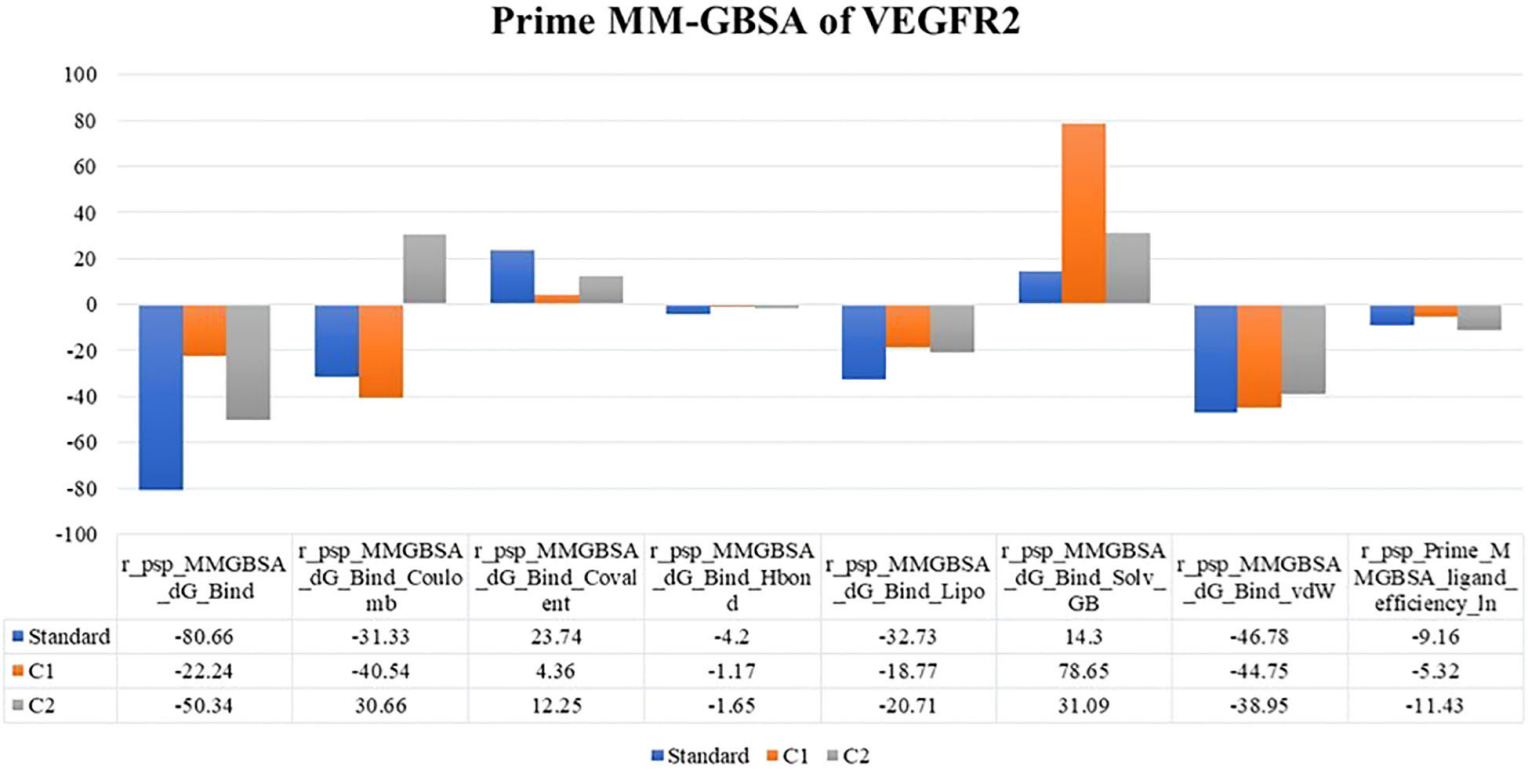
MM-GBSA binding energy values of the top two compounds along with the standard docked to VEGFR2.

The binding free energy for the two docked complexes in comparison with the standard was estimated *via* the Prime module of the Schrodinger suite. By examining the binding free energy values (−ΔG) (kJ/mole) of the best docked complexes, it was also possible to discern the importance of the ΔG_Bind Coulomb_ (Coulomb energy), ΔG_Bind Covalent_ (covalent binding energy), ΔG_Bind Hbond_ (hydrogen bonding correction), ΔG_Bind Lipo_ (lipophilicity energy), ΔG_Bind Solv GB_ (generalized Born electrostatic solvation energy) and ΔG_Bind vdW_ (van der Waals energy) in the respective complex stability. The results are summarized in tabular and graphical formats in **Figure 3**. Compared with labetalol (C1), risperidone (C2) had the closest values to the standard. This compound was further used for experimental anticancer assays.

### 3.4 Detection and purification of the risperidone compound *via* HPLC

The HPLC method was adopted to develop and validate the simultaneous detection and quantitation of known risperidone compounds from the Risperdal tablet. Acetonitrile, water, and formic acid (50:50:0.2 v/v/v) under gradient conditions composed the modified mobile phase used for the HPLC separation of the risperidone component from the peak of the Risperdal tablet. Two chromatographic peaks were detected and separated. The risperidone compound was separated at a retention time of 1.63 at 275 nm, as shown in **Supplementary Figure S6**. The other peak was considered impure, with a retention time of 3.28. As our major concern is to isolate only the risperidone compound, to remove the impurities from the compound, we again run the HPLC to obtain a single linear peak for risperidone, as shown in **Supplementary Figure S6**.

### 3.5 LC Ms/Ms analysis of the risperidone compound

Using electrospray ionization, LC–MS/MS analysis was carried out in positive ion mode. At 411.243 m/z, a peak was found that corresponded to the risperidone compound that had been purified by HPLC confirming compound identity and >95% purity (**Supplementary Figure S7**). This peak was caused by the drug’s side chain ether linkage cleavage and subsequent removal of the hydroxyl group.

### 3.6 Risperidone inhibits proliferation and induces apoptosis in a panel of cancer cells

**Figure 4:**
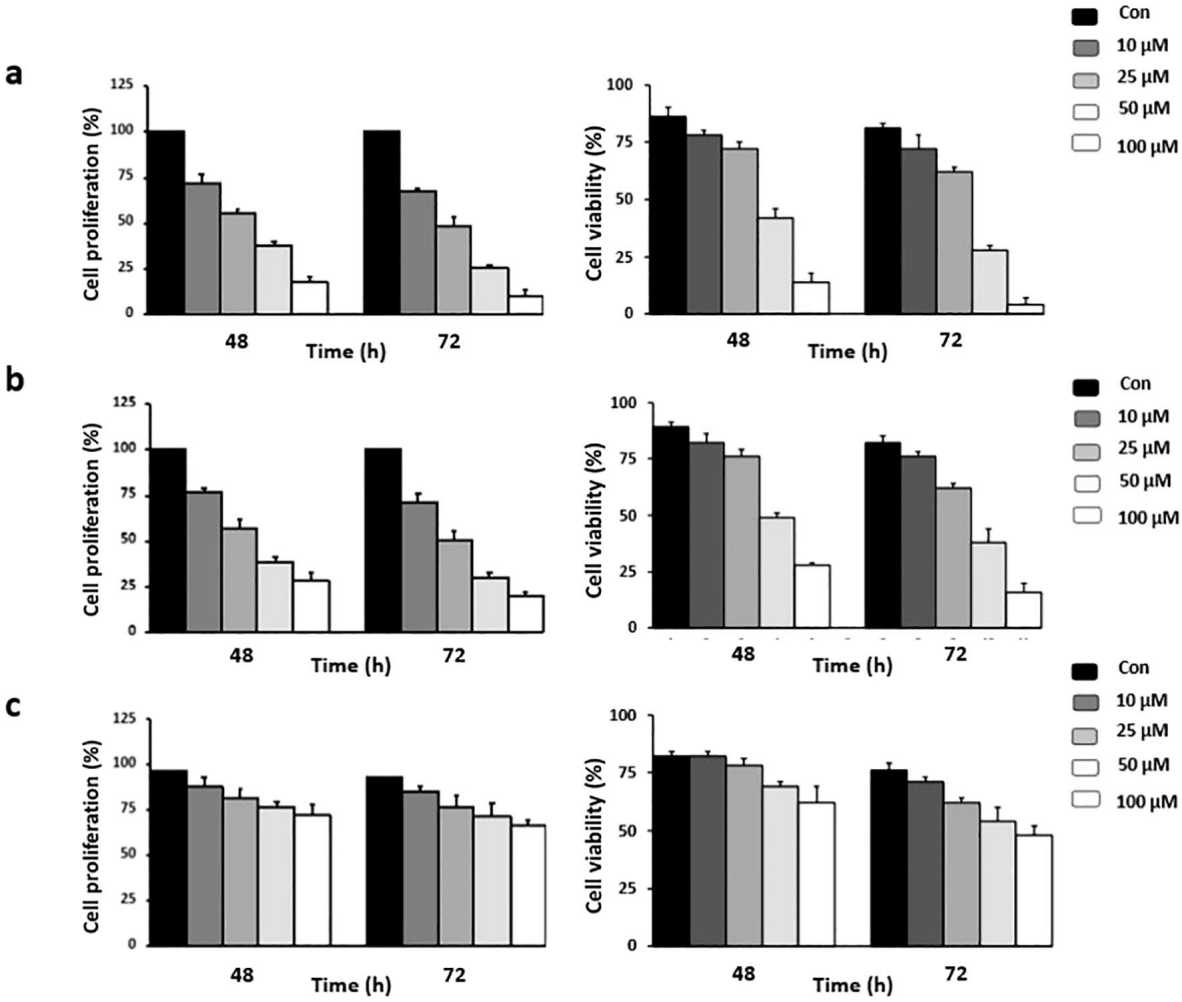
Effect of risperidone on cancer cell proliferation: Cells were treated with increasing concentrations of risperidone, incubated for 48 and 72 h, and subjected to MTT and Trypan blue dye exclusion assays to assess its cytotoxic potential. DMSO-treated wells served as vehicle controls. The experiments were conducted using **(A)** MDA-MB-468, **(B)** MDA-MB-231, and **(C)** HEK cells. Each experiment was repeated a minimum of 3 times. The error bars indicate the SEM, and P values were calculated by comparing the mean of the control group with the mean of the treated group (p <0.05).

Risperidone, an atypical antipsychotic drug used to treat schizophrenia and bipolar disorder, displays potent antiproliferative potential against various cancer cells. Treatment with risperidone efficiently diminished the proliferative potential of cells, as revealed by the MTT assay. The cell lines were treated for 72 h. The range of doses used was 10, 25, 50 and 100 μM. The results revealed that risperidone was effective against triple-negative breast cancer, with IC50 values ranging from 46.53 to 49.76 μM (**Figure 4**). Similar micromolar activities have been reported for other antipsychotics repurposed in TNBC models (43, 44). Risperidone treatment of cancer cells drastically reduced the percentage of viable cells, as demonstrated by the trypan blue dye exclusion assay, which further revealed the potent cytotoxic potential of risperidone.

### 3.7 Risperidone promotes apoptosis in cancer cells

**Figure 5:**
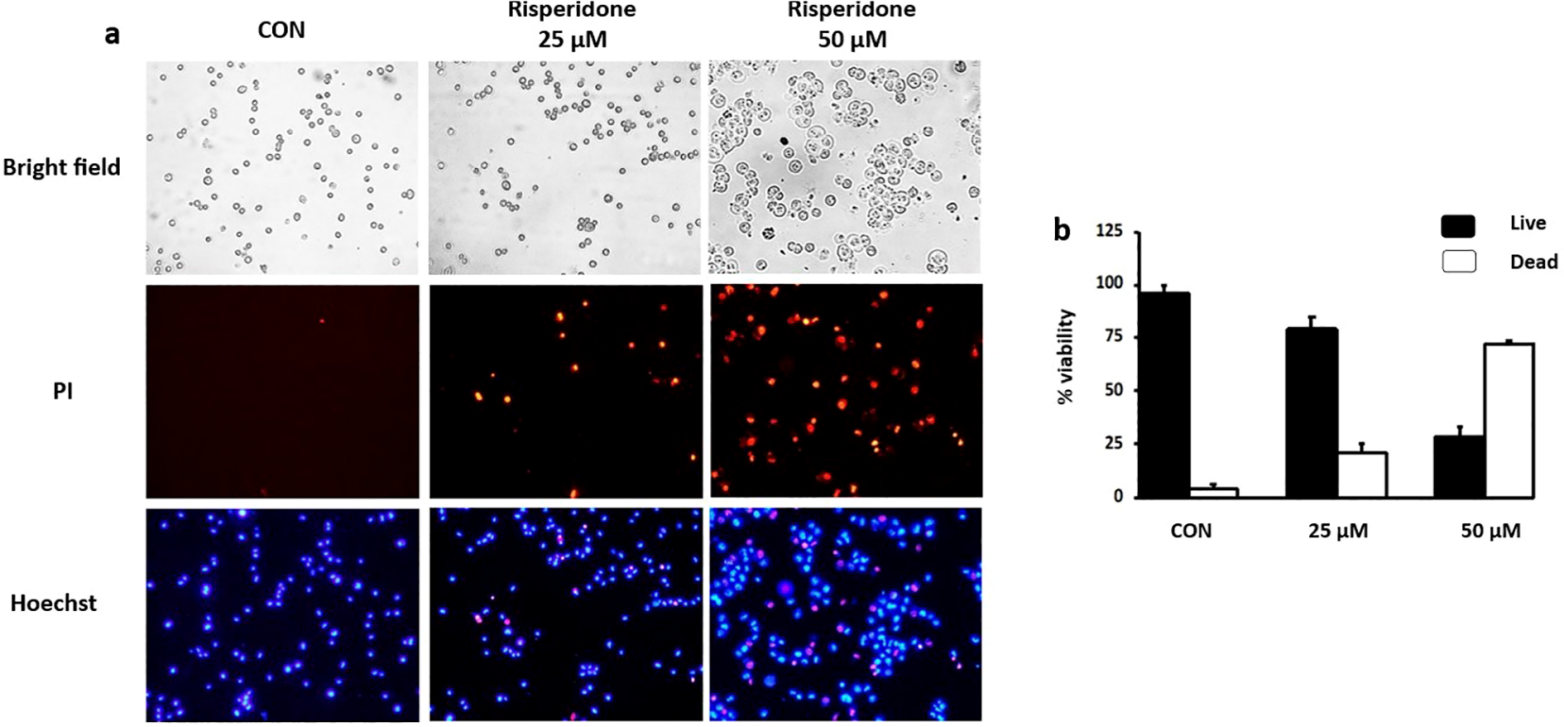
Effect of risperidone on apoptosis induction: **(a, b)** MDA-MB-468 cells were seeded into 6-well plates, treated with increasing concentrations of risperidone, incubated for 48 h and stained with Hoechst/PI. DMSO-treated wells served as vehicle controls. Scale bar: 50 µM.

Apoptosis is a natural process of programmed cell death that is crucial for maintaining normal tissue homeostasis. It can be triggered by various external or internal signals. Risperidone induces apoptosis in a concentration-dependent manner by targeting aberrantly overexpressed VEGFR in cancer cells, as demonstrated by Hoechst/PI dual staining (**Figure 5**). Risperidone significantly increased apoptotic cell death in a dose-dependent manner, with viability decreasing to 77 ± 6% (25 µM) and 28 ± 5% (50 µM) (n = 3 biological replicates, one-way ANOVA, p < 0.01).Morphological alterations in the cell nuclei were identified *via* fluorescence microscopy. The condensed nuclei of apoptotic cells are stained with blue, fluorescent Hoechst stain, a cell-permeable nucleic acid dye that is typically used to detect chromatin condensation and disintegration. Propidium iodide, a reddish-fluorescent dye that binds to DNA and causes cell damage, can stain cells only when there is a loss of plasma membrane integrity and an increase in plasma membrane permeability. The results clearly revealed the uptake of propidium iodide by MDA-MB-468 cells undergoing apoptosis, revealing the cytotoxic effect of risperidone on the screened cancer cells in a concentration-dependent manner.

### 3.8 Risperidone decreases colony formation

The formation of these colonies allows cancer cells to grow aberrantly, invade nearby tissues, and eventually metastasize to other parts of the body.

The effect of risperidone on colony formation was assessed *via* a focus formation assay. The results revealed a drastic decrease in the number of colonies formed by the treated cells compared with those formed by the DMSO-treated cells (**Supplementary Figure S8**). Colony numbers decreased from 152 ± 12 (control) to 42 ± 8 (treated) colonies per well. These results clearly demonstrate the potent antiproliferative potential of risperidone against the screened cancer cells.

### 3.9 Risperidone promotes mitochondrial dysfunction and induces apoptosis

**Figure 6:**
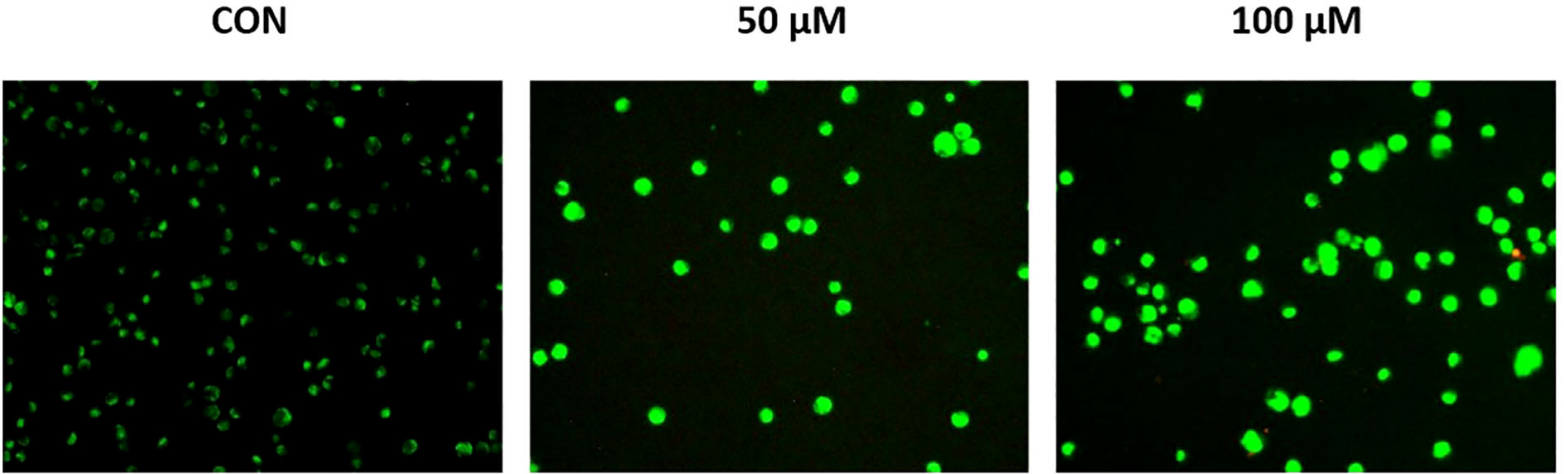
Effect of risperidone on mitochondrial membrane potential. MDA-MB-468 cells were seeded and treated with increasing concentrations of Risperidone, incubated for 24 h, harvested, and subjected to an MMP assay according to the manufacturer’s protocol. DMSO-treated cells served as the vehicle control. Scale bar: 200 µM.

The effect of risperidone on the mitochondrial membrane potential was evaluated *via* JC-1 dye. The JC-1 dye has an inherent cationic potential and aggregates in the mitochondrial lumen, resulting in red fluorescence. Induction of apoptosis results in disruption of the mitochondrial membrane, causing a change in the membrane potential and the accumulation of monomeric JC-1 dye in the cytosol, leading to green fluorescence. Treatment with risperidone efficiently induced apoptosis in a concentration-dependent manner by altering the membrane potential (**Figure 6**). The JC-1 red/green fluorescence ratio decreased from 1.00 ± 0.08 in control cells to 0.46 ± 0.05 at 50 µM and 0.31 ± 0.04 at 100 µM.

### 3.10 Anti-angiogenic assessment of risperidone

**Figure 7:**
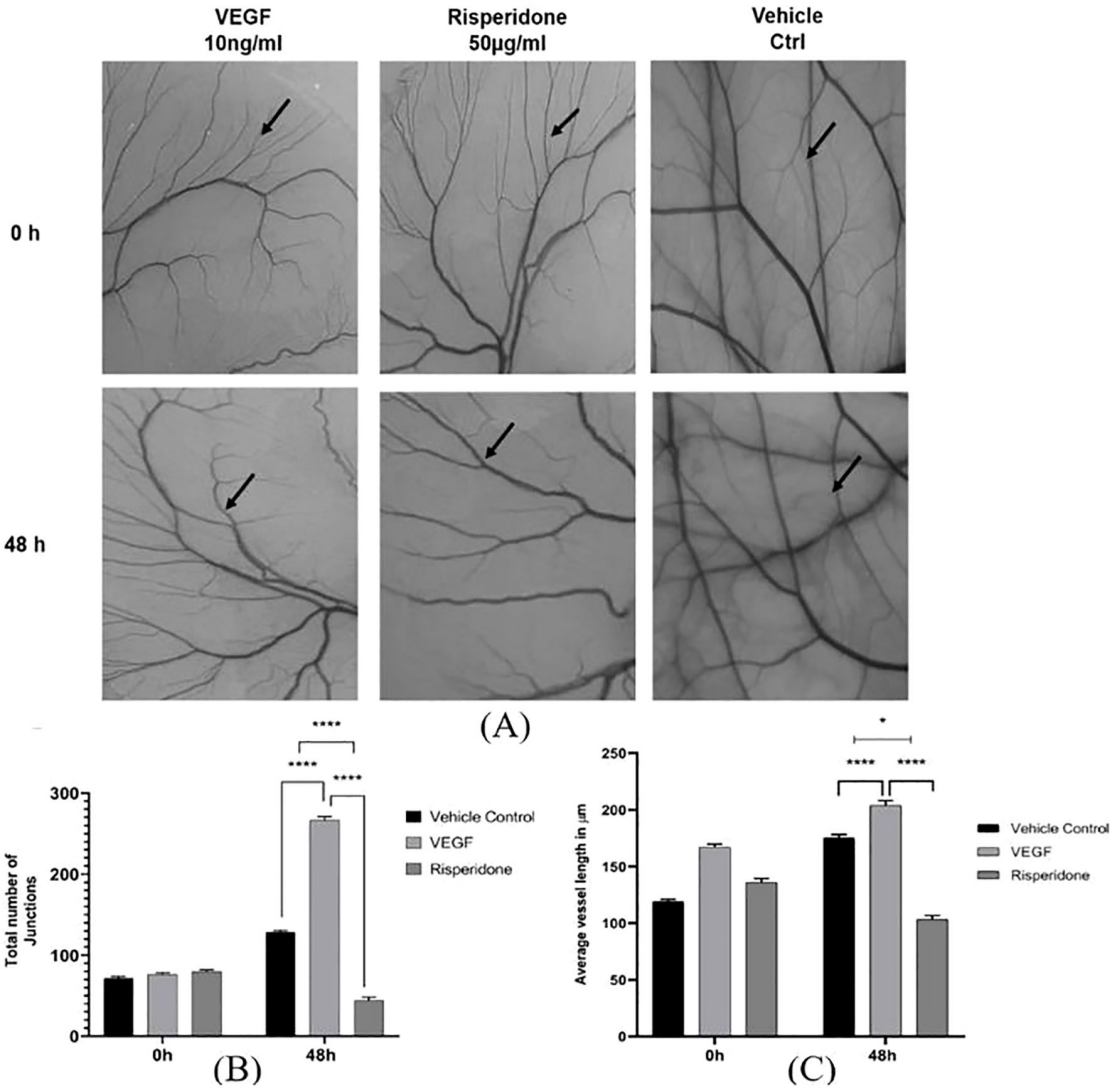
Effect of risperidone on the *in ovo* CAM assay: **(A)** Images showing the effects of treatment with risperidone (50 μg/mL) on CAM, vehicle control treatment on CAM and 10 ng/ml VEGF treatment on CAM at two different time intervals (0 h and 48 h). Analysis of blood vessel variation in terms of **(B)** total number of junctions and **(C)** average tubule length was noted, and the changes in CAM were represented in micrometers as computed from Angiotool software in comparison with the vehicle control. The values are presented as the means ± SDs, n=3. **** p<0.01 and *p<0.05 vs Vehicle ctrl. *p<0.05 vs 10 ng/mL VEGF. Scale bar = 200 µm; 4× magnification.

One of the most accurate assays for assessing angiogenesis is the chick chorioallantoic membrane (CAM) assay. The antiangiogenic property of risperidone was assessed *via* the CAM model by evaluating the total number of junctions and average vessel length. Images were captured and the number of branching points and length of blood vessels were quantified using the AngioTool Software. All extracted images have the same size and magnification with unified AngioTool inputs. As shown in **Figure 7**, the drug was administered to the CAM, and a significant effect was observed 48 h later at a single concentration of 50 μg/ml compared with the DMSO-treated controls and the VEGF-treated CAM models. Quantitative analysis showed that at 48 h, the total number of vascular junctions decreased by 46 ± 7% and average vessel length decreased by 42 ± 6% compared with VEGF-treated controls.

## 4 Discussion

Risperidone is a benzisoxazole derivative and an antipsychotic drug used to treat bipolar disorder, schizophrenia, or irritability associated with autistic disorder (45, 46). There are reports indicating its anticancer effect on gastric cancer (47) and colorectal cancer (48). Antipsychotic drugs also seem to enhance the effects of chemotherapy treatments such as doxorubicin and TMZ, overcoming tumor resistance to both chemotherapy and radiotherapy. These compounds seem to be promising anticancer agents on the basis of this mounting body of evidence, their accessibility and prior approval by the FDA, and other factors. A few reports also suggest that these antipsychotics exhibit antitumor and antimetastatic effects and may be used to treat TNBC alongside conventional chemotherapy (43, 44, 49). TNBC remains difficult to treat because it lacks the hormone receptors and HER2 targets that guide most breast-cancer therapies.

Structure-based drug repurposing offers a way to accelerate discovery by identifying approved agents with novel anticancer activity. In this study, integrated computational docking, MD simulations, *in vitro* assays, and in ovo CAM analysis together provide early evidence that a well characterized compound exerts anti-angiogenic and pro-apoptotic effects in TNBC models. These combined approaches bridge in silico predictions with functional outcomes and demonstrate the potential of repurposing centrally acting drugs to address an urgent therapeutic gap in TNBC.

Our data showed stable binding of the compound to the VEGFR2 ATP-binding site and a consistent reduction in proliferation and angiogenesis across MDA-MB-231 and MDA-MB-468 cells and the CAM model. Although VEGFR2 inhibition is a plausible mechanism, the link to apoptosis is likely multifactorial. Antipsychotics such as trifluoperazine and thioridazine have been reported to induce G0/G1 arrest, mitochondrial membrane depolarization, and ROS-mediated apoptosis in TNBC models (43, 44). Similar pleiotropic effects are known for other psychotropics, including modulation of dopamine and serotonin receptors and interference with mitochondrial bioenergetics (49). The pro-apoptotic activity observed here may therefore arise from a convergence of VEGFR2 blockade with off-target signaling events, underscoring the need for mechanistic validation beyond receptor docking.

This work also extends prior observations of antipsychotic anticancer activity by integrating multiple experimental platforms. Earlier reports noted risperidone’s context-dependent effects in gastric and colorectal cancers (47, 48), but none combined molecular modeling, cytotoxicity assays, and CAM-based angiogenesis testing in TNBC. Compared with established VEGFR2 inhibitors such as sunitinib or bevacizumab, the micromolar IC_50_ values observed here indicate lower potency, yet they are within the range reported for other repurposed antipsychotics and may still hold translational value, particularly for patients at risk of brain metastases given the drug’s known CNS penetration (50, 51). These findings position this compound as a candidate for combination regimens rather than as a stand-alone anti-angiogenic therapy.

Several limitations temper the interpretation of our results. Protein-level confirmation of VEGFR2 inhibition was not performed, the CAM assay used a single concentration without a pharmacological positive control, and systematic toxicity studies were outside the present scope. Because the compound is psychoactive with known central nervous system adverse effects (45, 46), strategies such as dose optimization, targeted delivery, or nanoparticle formulations will be essential to mitigate potential toxicity in oncology settings. Future studies should incorporate dose–response CAM assays, *in vivo* TNBC models, and combination strategies with standard chemotherapies to define clinically achievable exposures and clarify mechanism of action.

Compared with other reports of antipsychotics in oncology, our study provides a broader integration of computational and functional data while highlighting critical translational hurdles. Trifluoperazine and thioridazine, for example, have demonstrated nanomolar activity but limited clinical advancement due to cardiotoxicity and neurological effects; risperidone shows weaker potency yet benefits from a well-defined safety profile and pharmacokinetics from decades of psychiatric use. By acknowledging the relatively high IC_50_ values, the absence of direct VEGFR2 phosphorylation data, and the need for targeted delivery to avoid CNS toxicity, this work frames risperidone as a starting point for combination regimens or brain-metastasis prophylaxis rather than as a single-agent therapy. The CAM assay is a refined model for animal research since it causes minimal damage to chick embryos, in contrast to other *in vivo* models, such as mouse subcutaneous implants (52). Taken together, the present findings establish a proof-of-concept that supports risperidone’s repositioning potential in TNBC while underscoring the need for further mechanistic, quantitative, and translational studies to determine its true therapeutic relevance.

## 5 Conclusion

The findings of our study demonstrate that risperidone can hinder breast cancer angiogenesis *in silico, in vitro* and *in ovo* through its anti-VEGFR2 activity and could serve as a novel repurposed agent for TNBC therapy, warranting further preclinical and clinical studies. These data are exploratory and require confirmation in animal studies and detailed pharmacokinetic-pharmacodynamic analyses before any clinical consideration. However, the limitations of the present study include investigating potential synergistic effects with chemotherapy, evaluating off-target effects, and determining the clinical prescription status of risperidone as a repurposed drug. Future studies will address and examine the current constraints and limitations of the study to improve the comprehensiveness of our research. Nonetheless, it is important to note that our research is limited to *in vitro* experiments and *in ovo* antiangiogenic assays, and further investigations are warranted to assess the impact of risperidone on TNBC *in vivo in detail* before clinical translation.

## Data Availability

The original contributions presented in the study are included in the article/**Supplementary Material**. Further inquiries can be directed to the corresponding authors.
