# Supplementary material for: Repurposing risperidone as an anti-angiogenic agent for triple-negative breast cancer: a computational to in ovo investigation
Source: Front Oncol. 2025 Oct 28;15:1645905. doi: 10.3389/fonc.2025.1645905 (PMC12602248; doi:10.3389/fonc.2025.1645905)
Supplement: Supplementary file 1 [file DataSheet1.docx]

Supplementary Material


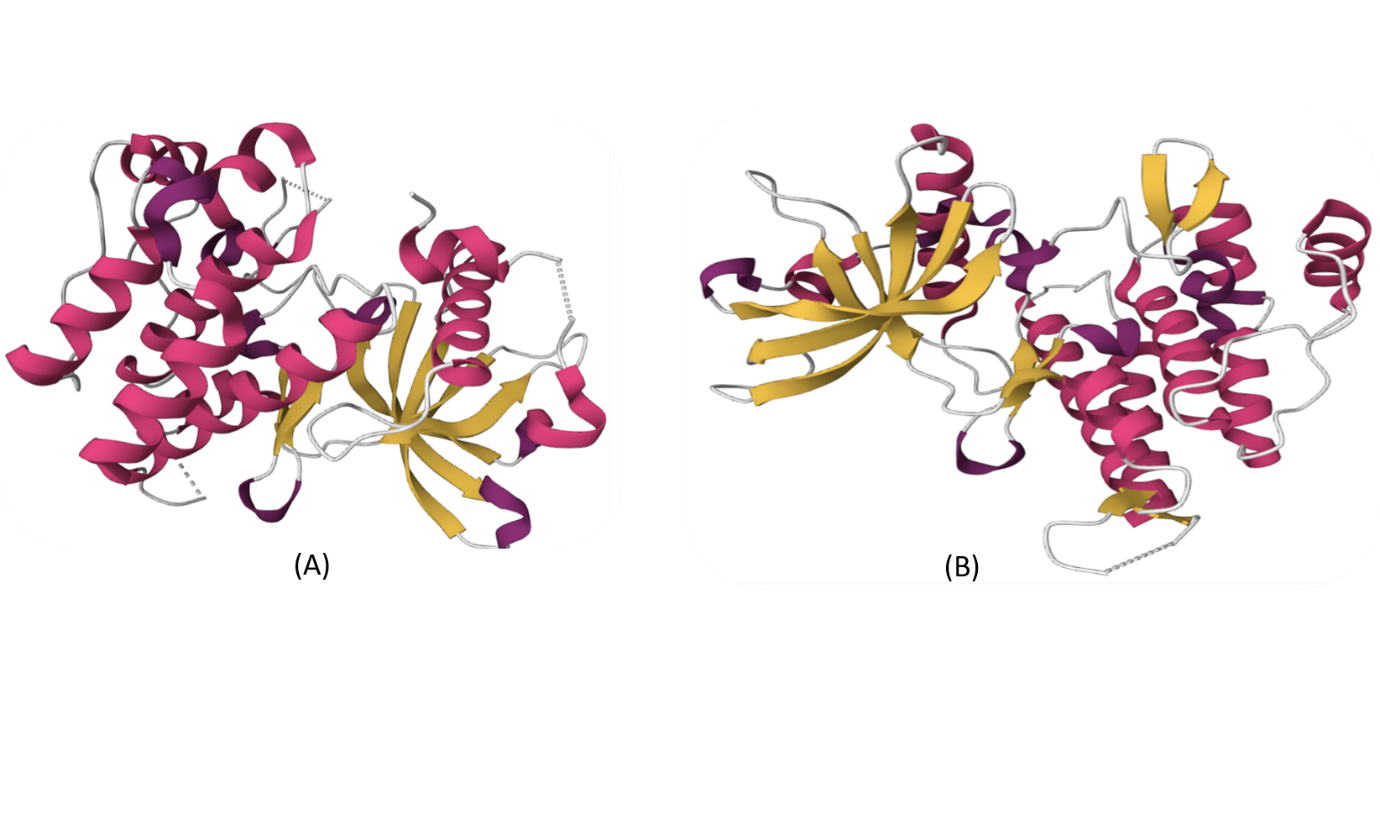


**Supplementary Figure 1.** Crystal structures of (A) VEGFR1 (3HNG) (B)VEGFR2 (4AG8).


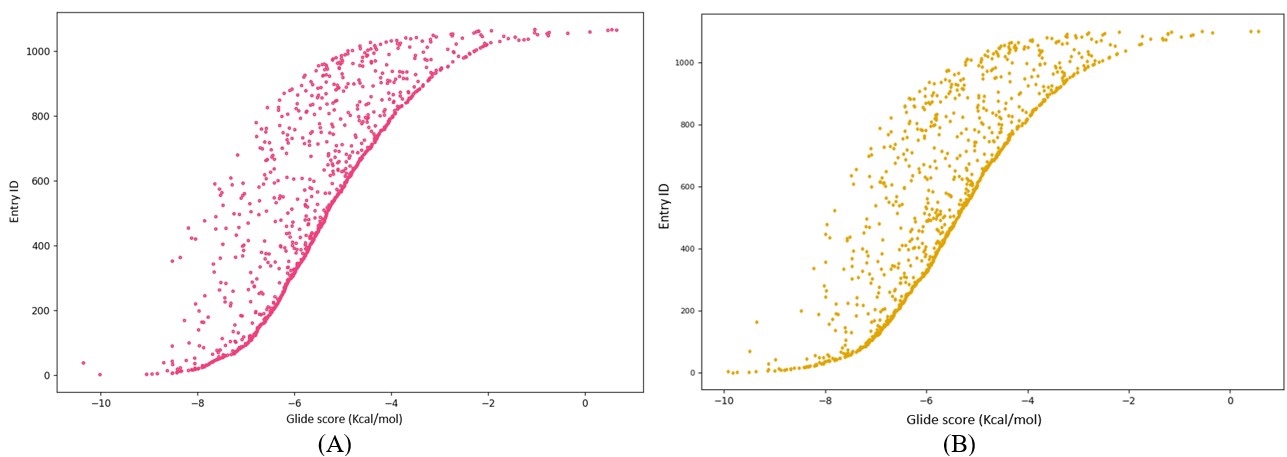


**Supplementary Figure 2.** A graphical representation of glide score (Kcal/mol) of FDA approved compounds with (A) VEGFR1 and (B) VEGFR2 wherein each dot in the graph represents a ligand.


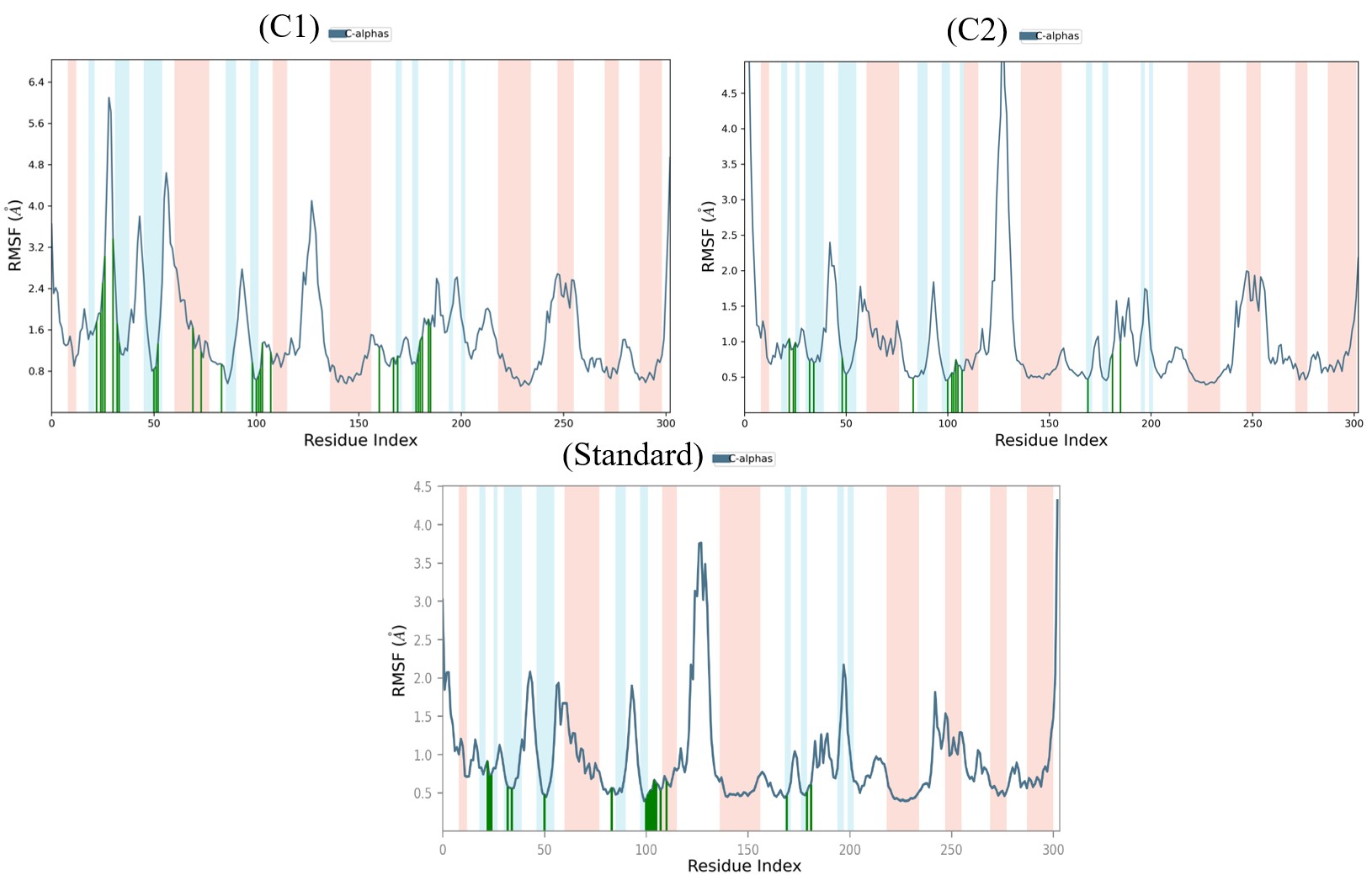


**Supplementary Figure 3.** The Protein RMSF plots of labetalol (C1), risperidone (C2) and standard in complex with VEGFR2.


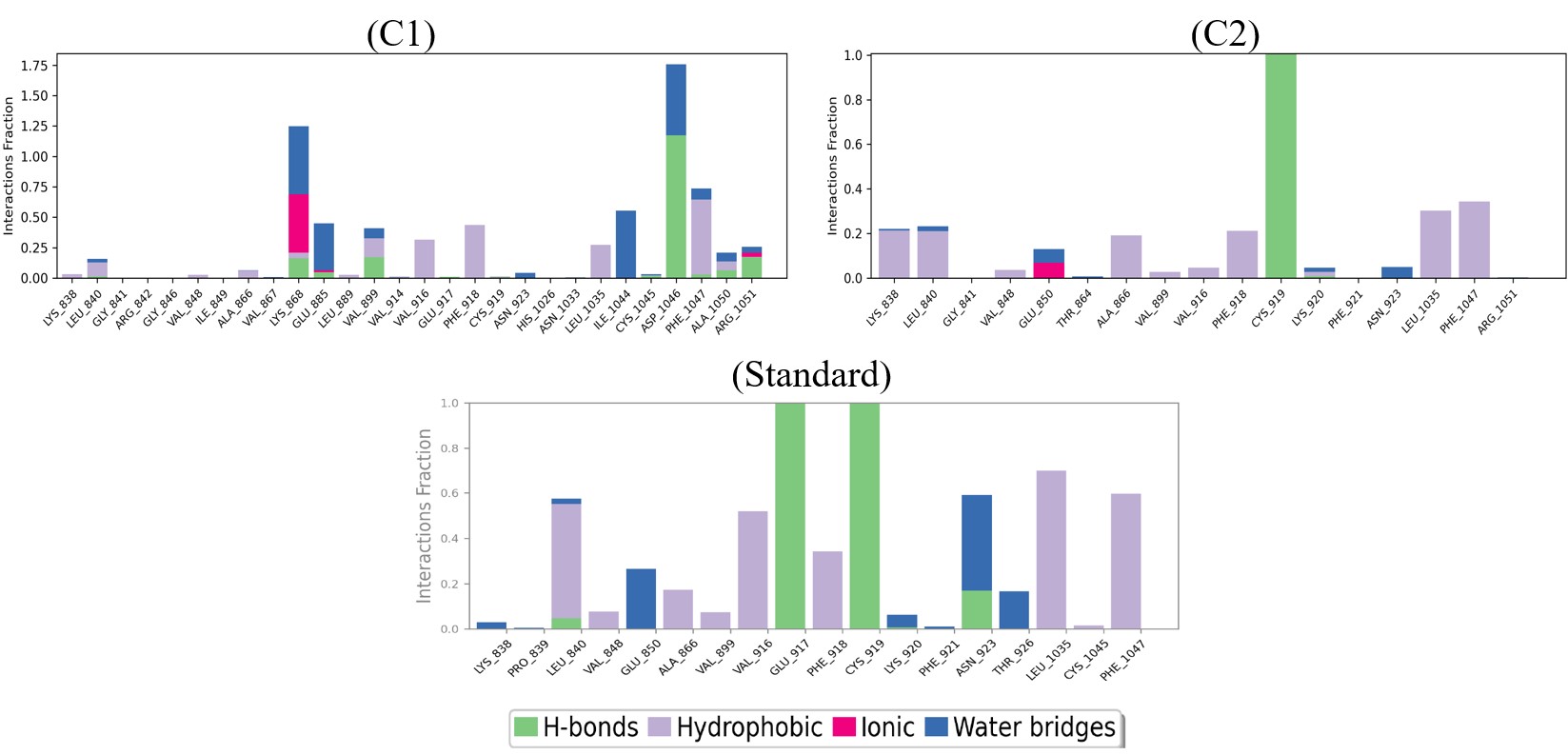


**Supplementary Figure 4.** Protein-ligand interactions of VEGFR2-labetalol (C1), VEGFR2-risperidone (C2) and VEGFR2-Standard complexes categorized in four different types : Hydrogen Bonds, Hydrophobic, Ionic and Water Bridges.


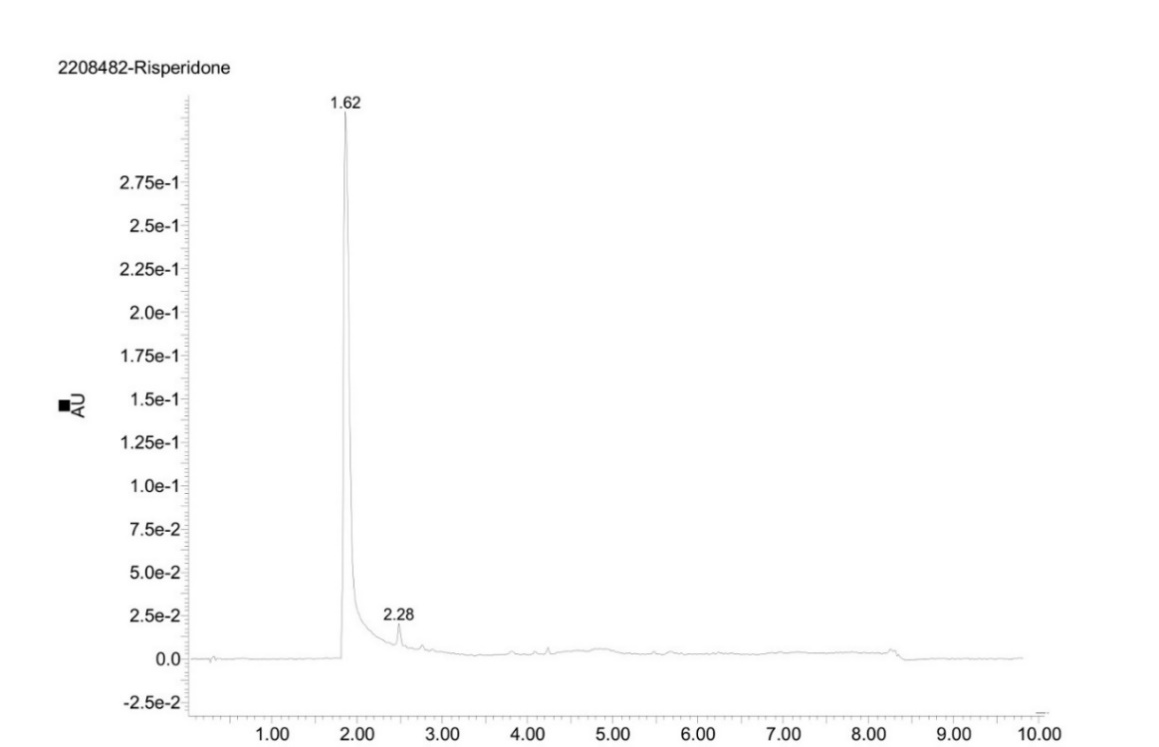


**Supplementary Figure 5.** Chromatogram of a Risperdal tablet, representing two peaks, Peak 1 is risperidone compound, tR:1.63 min and peak 2 is unknown, tR: 3.28 min.


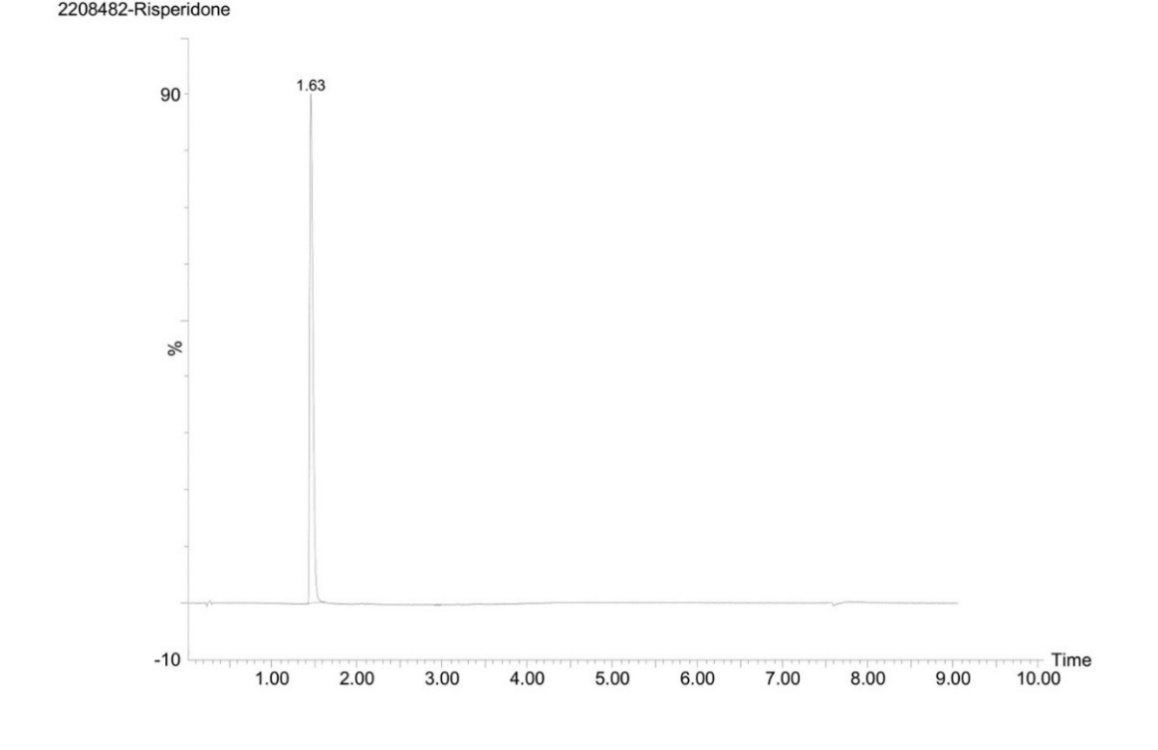


**Supplementary Figure 6.** Chromatogram of a purified risperidone compound peak at tR: 3.28 min.


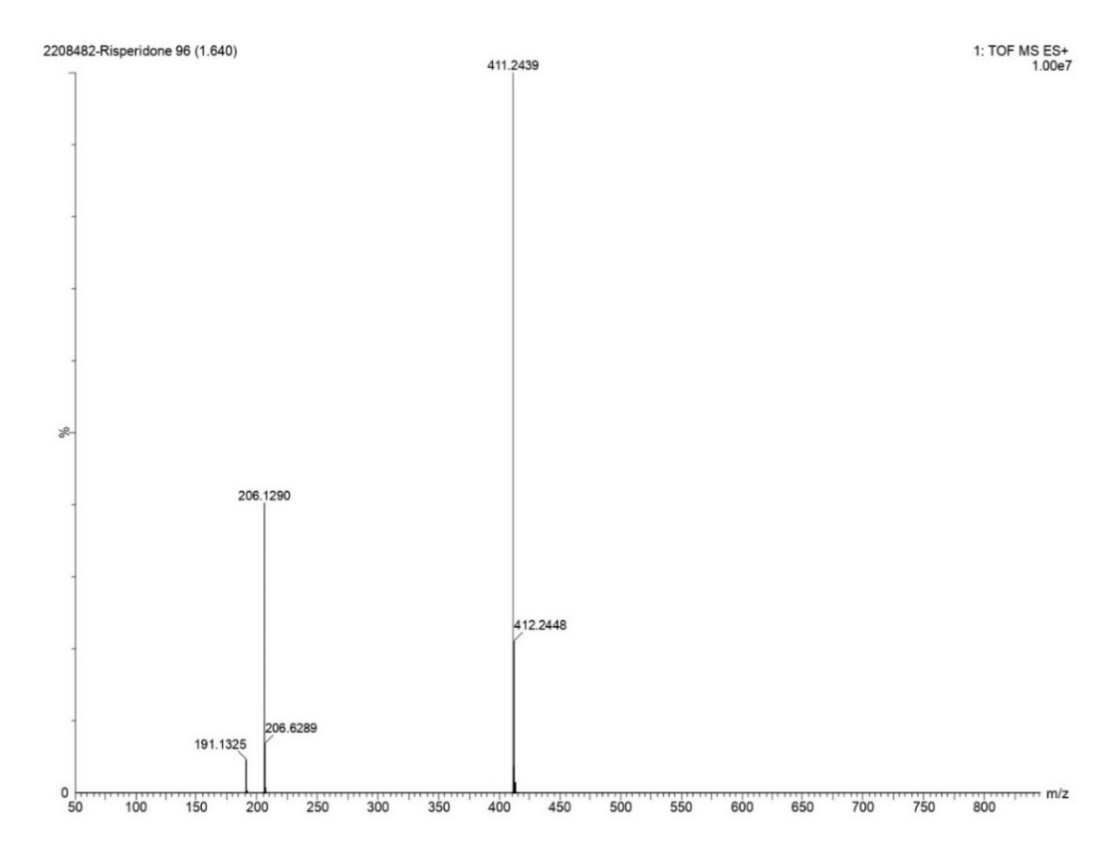


**Supplementary Figure 7.** Mass spectrum showing the ESI Positive ion mode scan of HPLC purified peak at 411.243 m/z at [M+H]+ 3.28 min.


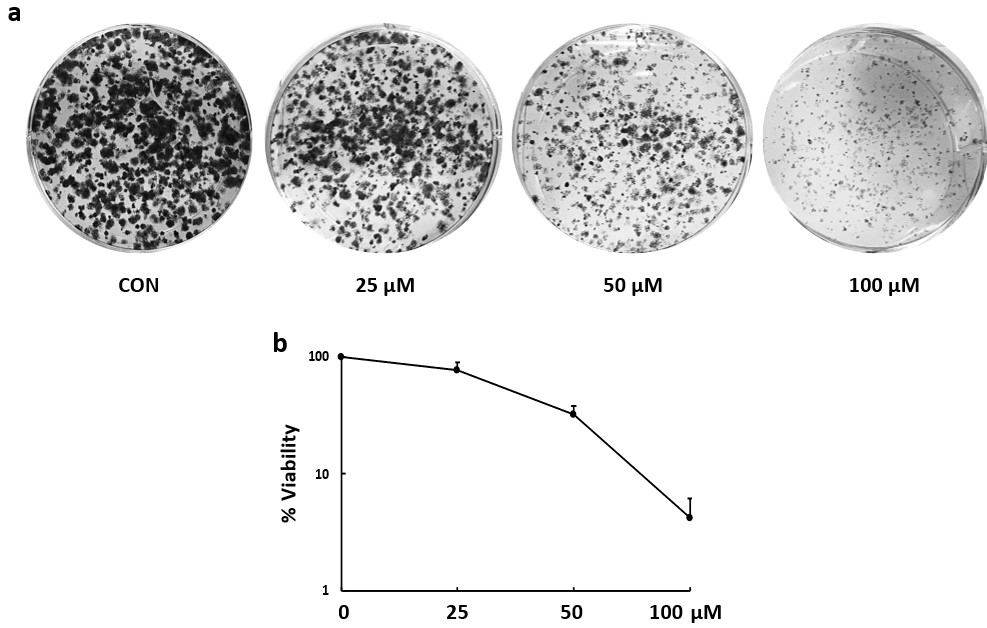


**Supplementary Figure 8. Effect of Risperidone on Colony formation:** Cells were seeded into a 6-well plate and treated with increased concentrations, allowed for 2 weeks to form colonies, and stained with crystal violet. (a) MDA-MB468 cells, (b) bar graph. DMSO-treated wells served as the vehicle control; each experiment was repeated 3 times.
